# RNA Structures and Their Role in Selective Genome Packaging

**DOI:** 10.3390/v13091788

**Published:** 2021-09-08

**Authors:** Liqing Ye, Uddhav B. Ambi, Marco Olguin-Nava, Anne-Sophie Gribling-Burrer, Shazeb Ahmad, Patrick Bohn, Melanie M. Weber, Redmond P. Smyth

**Affiliations:** 1 Genome Architecture and Evolution of RNA Viruses, Helmholtz Institute for RNA-Based Infection Research, Helmholtz-Center for Infection Research, 97080 Würzburg, Germany; liqing.ye@helmholtz-hiri.de (L.Y.); uddhav.ambi@helmholtz-hiri.de (U.B.A.); marco.olguin@helmholtz-hiri.de (M.O.-N.); anne-sophie.gribling@helmholtz-hiri.de (A.-S.G.-B.); shazeb.ahmad@helmholtz-hiri.de (S.A.); patrick.bohn@helmholtz-hiri.de (P.B.); melanie.weber@helmholtz-hiri.de (M.M.W.); 2 Faculty of Medicine, University of Würzburg, 97080 Würzburg, Germany

**Keywords:** RNA virus, RNA, RNA structure, genome packaging, viral assembly, evolution

## Abstract

To generate infectious viral particles, viruses must specifically select their genomic RNA from milieu that contains a complex mixture of cellular or non-genomic viral RNAs. In this review, we focus on the role of viral encoded RNA structures in genome packaging. We first discuss how packaging signals are constructed from local and long-range base pairings within viral genomes, as well as inter-molecular interactions between viral and host RNAs. Then, how genome packaging is regulated by the biophysical properties of RNA. Finally, we examine the impact of RNA packaging signals on viral evolution.

## 1. Introduction

Genome packaging is the process whereby viruses assemble their genomes into capsids [1]. The primary purpose of the capsid is to protect the genome from a hostile cellular and extracellular environment until its cargo can be released into a new host for a further round of replication. For faithful replication it is essential that genome packaging occurs with high fidelity. In the case of RNA viruses, this is particularly challenging because viral RNA must be specifically selected from a complex mix of cellular RNA, which often includes non-genomic viral RNA. Furthermore, genome packaging must be tightly regulated as it is often in competition with other essential functions, such as genome replication or translation. RNA viruses have solved this problem by exploiting the capacity of RNA to fold into three-dimensional structures that are recognised by viral packaging machinery [2,3,4,5,6,7,8]. RNA structures are formed from local intra-molecular and long-range base pairings within the same molecule, as well as inter-molecular RNA-RNA interactions [9]. Because RNA structures are rarely static, packaging can be dynamically regulated by intrinsic RNA structural switches, binding of viral factors, or in some cases, inter-molecular interactions with host RNAs.

Interestingly, many RNA viruses have evolved genome organizations that greatly complicate viral assembly and packaging. Segmented viruses, such as influenza and rotavirus, need to incorporate multiple genome segments for their virions to be infectious [10]. On the other hand, retroviruses package two copies of their genome, even though the total genetic material of only one genome is replicated [11]. In exchange for this increased complexity, RNA viruses enhance their evolvability through recombination or reassortment. Here, genome packaging uses specific inter-molecular RNA interactions that bring together different segments or genomes for assembly. In this review, we discuss how RNA viruses exploit the properties of RNA structure to regulate their packaging and explain how RNA based packaging mechanisms can influence viral evolution.

## 2. Packaging Signals in RNA Viruses

**Figure 1:**
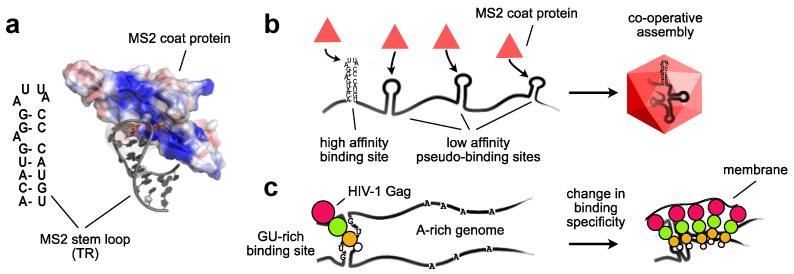
RNA packaging signals (**a**) MS2 bacteriophage encodes a coat protein (CP) that binds to a 19-nucleotide stem loop structure, known as TR. Secondary structure model of the TR stem loop and three-dimensional structure of the coat protein-TR interaction; (PDB: 1AQ3) (**b**) MS2 bacteriophage genome is encapsidated through cooperative interactions between coat protein that are bound to a high affinity binding site (TR) and multiple low affinity binding sites encoded throughout the genome; (**c**) HIV-1 Gag recognizes the genomic RNA through a GU-rich high affinity binding site in the 5′ untranslated region (5′UTR). During assembly at the plasma membrane Gag switches its specificity from GU-rich sequences to A-rich sequences, which is proposed to favour assembly of Gag on its cognate genomic RNA.

RNA viruses distinguish their genomes from cellular RNAs using *cis* acting packaging signals that serve as high affinity binding sites for the viral capsid (or nucleocapsid) proteins [12]. RNA readily folds back on itself to form secondary and tertiary structures, which are complex enough to enable the specific recognition of RNA by proteins [13]. A canonical example is the 19 nucleotide stem-loop, also known as TR, in the genome of the MS2 bacteriophage (Figure 1a) [14,15,16]. The MS2 coat protein (CP) dimer specifically recognizes this short stem loop structure to initiate assembly. Despite the simplicity of the structure, which is only formed from local base-pairings, MS2 CP binds with high affinity and specificity. This has led to the extensive repurposing of the TR-CP interaction in applications such as single molecule live cell imaging [17,18]. Remarkably, stem-loops having a C at position −5 in the loop have a higher affinity for CP than the wild-type U [19]. Whilst this is useful for biotechnology purposes, it also neatly demonstrates that increased affinity is not always beneficial for viruses, presumably because genomes must eventually be released during the early steps of the next replication cycle. Interestingly, high-throughput RNA structure-function analyses reveal that nucleotides in the stem can be exchanged without impairing binding to CP. In contrast, specific single stranded residues were required for function [20,21]. This is because, for the most part, RNA binding proteins (RBPs) make non-specific interactions with double stranded RNA (dsRNA) through generic contacts with the 2′hydroxyl groups of the ribose or the phosphodiester backbone [22]. For RNA viruses, non-specific affinity is usually driven by electrostatic interactions between positively charged patches on capsid protein subunits and the negatively charged nucleic acid genome. On the other hand, unique structural features created by loops and mismatches can be more readily recognized through extensive sequence-specific contacts [23].

MS2 bacteriophage genome packaging depends not only on the high affinity TR interaction site, but also on lower affinity pseudo-packaging sites that are dispersed throughout the genome (Figure 1b) [16,24]. This strategy is proposed to enhance the specificity and efficiency of assembly through cooperative interactions with viral capsid proteins [25]. This can be seen in other RNA viruses, such as tobacco necrosis virus (TNV) [26], hepatitis B virus (HBV) [27], and alphavirus [28]. Moreover, similar features emerged in directed evolution experiments that successfully converted a bacterial enzyme into a nucleocapsid that packages and protects its own encoding mRNA [29]. On the contrary, HIV-1 has no described packaging signals outside of the 5′ end of the genome. Instead, assembly on viral genomic RNA is driven by changes in the specificity of the structural protein Gag during its multimerization at the plasma membrane [30] (Figure 1c). This change in specificity enhances its affinity for A-rich sequences that are enriched in the HIV-1 genome compared to cellular RNA [30,31]. The prevailing model is that specific and high affinity binding sites nucleate viral assembly, whereas lower affinity binding sites and/or non-specific interactions are needed to drive growth and assembly of the capsid structure from capsid subunits [25,32]. Indeed, under physiological conditions, electrostatic repulsion between positively charged capsid subunits inhibit assembly to prevent the wasteful formation of empty capsids. This repulsion is counteracted by electrostatic attraction between capsid subunits and the negatively charged RNA genome. Essentially, electrostatic interactions provide the thermodynamic driving force for capsid assembly, converting the reversible capsid protein(s)-genome interaction into an irreversible assembly reaction [33,34]. RNA may even be considered as a structural component of virions. In the absence of genomic RNA, retroviruses package cellular RNAs, and retroviral cores can be disrupted upon treatment with RNase [35]. Moreover, recent work with the non-enveloped brome mosaic virus (BMV) has shown that when capsid subunits assemble on small non-viral RNAs it forms unstable shells with non-icosahedral structures that may be rapidly recycled into icosahedral capsids when the genomic RNA becomes available [36]. Furthermore, impressive cryo-electron microscopy (cryo-EM) reconstruction of the MS2 bacteriophage demonstrate how distinct, but non-uniformly distributed RNA structures favour assembly of the correct icosahedral capsid structure over non-productive octahedral structures [16]. Evidently, optimal viral assembly depends on the biophysical properties of the genomic RNA, which in turn is driven by a myriad of factors, including RNA sequence, structure [37,38] and size [36].

**Figure 2:**
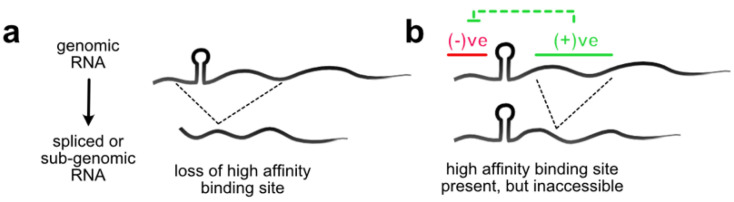
Selection of genomic RNA from non-genomic viral RNA. Dotted lines signify splicing or production of sub-genomic RNA. (**a**) Non-genomic RNA cannot be packaged because it does not contain the high affinity binding site for the (nucleo)capsid protein; (**b**) Non-genomic RNA contains the sequences for high affinity binding site, but it is not presented for binding. In HIV-1, negative regulatory elements (−ve) upstream of the splice donor (SD) conceal the Gag binding site unless counteracted by positive regulatory (+ve) sequences downstream of the SD.

Complex viruses that express sub-genomic or spliced viral RNAs have an additional challenge: they must not only distinguish their genomic RNA from cellular RNA, but also from non-genomic viral RNAs (Figure 2). One simple way to achieve this selectivity is the removal of the packaging signal from the non-genomic RNA during its production. This mechanism occurs in certain retroviruses, such as Moloney murine leukemia virus (MoMLV), which contains a packaging signal with high affinity binding sites for viral nucleocapsid (NC) composed of three stem-loop structures (DIS-2, SL-C and SL-D) [39,40]. All these RNA structures lie downstream of the major splice donor site and are thus removed from spliced viral RNAs (Figure 2a). Another retrovirus, HIV-1, recognizes its genomic RNA through specific interactions between the viral Gag protein and packaging signals present at the 5′ end of the genome [41,42,43,44,45,46,47]. Early deletion mutagenesis studies identified SL3 (Ψ), which lies downstream of the major splice donor SL2, as the major packaging motif [46,47,48]. This genome organization was originally thought to explain the selectivity for genomic over spliced viral RNA [49,50]. However, an abundance of evidence has now revised this picture. Specifically, the basal part and internal loop of SL1, which lies upstream of SL2, is now recognized as the primary Gag binding site [21,51,52,53,54]. Notably, deletion or mutagenesis of SL1 has a more drastic effect on Gag binding and genome packaging compared to SL3, and deletion of sequences downstream of SL2 has only modest effects on binding [52,53]. This revision in understanding resurrected the problem of how HIV-1 discriminates between spliced viral RNA and genomic RNA. Surprisingly, genome fragments from the first nucleotide through to SL3—containing SL1—are not efficiently bound by Gag unless they contain sequences downstream of SL3 [51]. A model was proposed whereby a long-range interaction between sequences downstream of the splice donor site counteracts a negative regulatory element upstream of the high affinity binding site in SL1 [51] (Figure 2b). As this interaction can only be formed in genomic RNA, it enables the selectivity of Gag for genomic RNA over spliced viral RNA at the initial binding step [51,54,55,56,57]. A good candidate for this long-range interaction is the so-called U5-AUG base pairing, which is thought to promote genome packaging (discussed in the next section). However, it is not excluded that additional structural features within the packaging region SL1 to SL3, which are present only in genomic RNA, collaborate to promote nucleation of the Gag-RNA complex [21,41,57,58].

**Figure 3:**
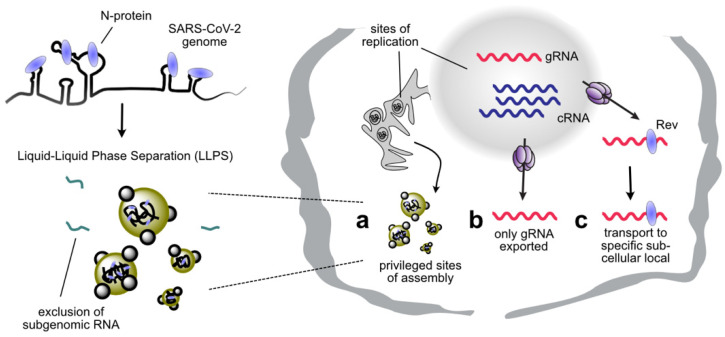
Successful genome packaging requires RNA signals to direct the genome from sites of replication to sites of assembly. (**a**) Sites of assembly generated by liquid-liquid phase separation (LLPS). Specific interactions between the SARS Coronavirus-2 (SARS-CoV-2) nucleoprotein (N) and its genome induce LLPS. Sub-genomic RNA is excluded. (**b**) Influenza complementary RNA (cRNA) replication intermediates are not correctly exported from the nucleus and are therefore not packaged. (**c**) HIV-1 genomic RNA contains the Rev Response Element (RRE) which binds to the viral Rev protein needed to export the RNA from the nucleus. Rev binding is proposed to enhance packaging by transporting RNA to the correct sub-cellular location.

Coronaviruses (CoV) have extraordinarily large genomes (~30 kb) that presumably pose additional difficulties for packaging, yet genomic RNA is efficiently and selectively incorporated into virions [5,59,60,61]. Accumulating evidence suggests that SARS-CoV-2 exploits liquid-liquid phase separation (LLPS) during its replication [62,63,64,65,66,67,68,69,70] (Figure 3). LLPS occurs when biological molecules condense into a phase resembling a liquid droplet, and is an emerging paradigm for organizing membrane-less viral factories [71,72]. It is a common property of RBPs containing intrinsically disordered regions (IDR), such as the SARS-CoV-2 nucleocapsid (N) [64,67,68,73]. LLPS of N protein is enhanced in the presence of viral RNA [62,65], and even though N protein binds throughout the genomic RNA [62,70], LLPS is specifically promoted by RNA sequences at the 5′ and 3′ of the SARS-CoV-2 genome (Figure 3) [62]. Interestingly, other sequences, such as the CoV frameshift site, were found to disperse condensates [62], and importantly, sub-genomic RNA was efficiently excluded from preformed droplets [62]. This demonstrates that for LLPS mediated packaging, the biophysical properties of RNA-protein interaction are as important as the protein-RNA affinities. LLPS likely promotes viral assembly by enhancing interaction between RNA and N protein within a privileged site [62], but may have other roles in viral replication, such as hiding viral RNA from cellular immune sensors [74,75].

Packaging sites may also include motifs necessary for the correct presentation of the RNA molecule in time and space. For example, influenza viruses have a segmented genome of negative sense viral RNAs (vRNAs) that are replicated in the nucleus via complementary RNA (cRNA) intermediates. Long-range interactions between the 5′ and 3′ termini, in some cases over distances of thousands of nucleotides, construct the promoter structure that is involved in transcription, replication and packaging [76]. Interestingly, cRNAs and vRNAs are both complexed into ribonucleoproteins (vRNPs) with very similar protein compositions, but only vRNPs are packaged into virions. Slight differences in promoter structures between vRNPs and cRNPs, due to imperfect complementarity between the terminal sequences, affect its interaction with the viral M1 protein that acts as a bridge between vRNPs and the nuclear export machinery. This structural difference allows the virus to discriminate between cRNPs and vRNPs by either preventing nuclear export of the cRNP [77] or by changing nuclear export pathways [78] (Figure 3). In the same vein, several studies show that the binding of the HIV-1 Rev protein to its cognate RNA structure, the Rev Response Element (RRE), enhances genome packaging [79,80,81]. Surprisingly, this enhancement effect seems to be unrelated to the role of Rev/RRE in increasing cytoplasmic RNA levels. Rather, the Rev/RRE is proposed to enhance packaging by defining the correct nuclear export pathway and subcellular localization of the genomic RNA [79,80,81,82] (Figure 3).

## 3. RNA Structure as a Regulator of Genome Packaging

Genome packaging occurs during the late stages of replication when sufficient genomes and structural proteins have been replicated and produced to ensure effective viral assembly. Sometimes even, the same viral RNA molecule must carry out several competing functions. It is not surprising therefore that viruses heavily regulate the translation, replication, and packaging of their genomes. RNA viruses achieve this, in part, by exploiting the dynamic and flexible properties of RNA. Namely, RNA molecules can spontaneously fold into multiple, mutually exclusive structures, acting as riboswitches with each structure having a different function [83]. RNA can also respond to the binding of cellular or viral biomolecules, which can act as a regulatory trigger for further remodelling of ribonucleoprotein complexes [84].

**Figure 4:**
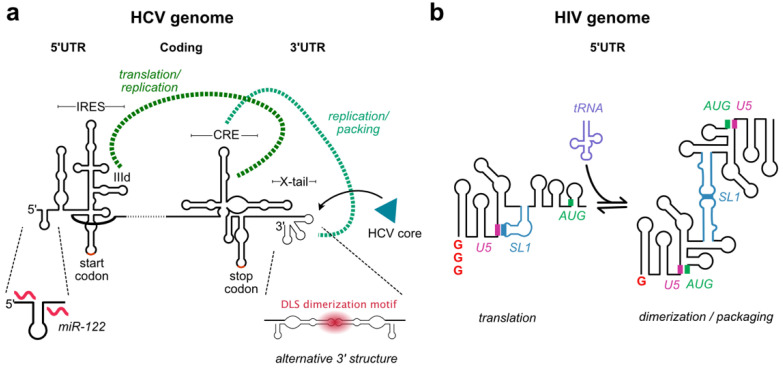
RNA structural switches and long-distance interactions regulate the balance between genome replication, packaging, and translation. (**a**) The HCV life cycle is regulated by a complex network of long-distance intra-molecular interactions and inter-molecular interactions. The packaging site which binds to the HCV core protein resides in the 3′ untranslated region (3′UTR). A long-distance base pairing between the *cis*-acting element (CRE) in the coding region and the X-tail in the 3′UTR regulates the balance between replication and packaging (light green dotted line). A long-distance interaction between the CRE and the internal ribosome entry site (IRES) regulates the balance between replication and translation (dark green dotted line). The 3′UTR is alternatively structured, leading to the formation of homodimers through an intermolecular interaction. The HCV 5′UTR binds the host microRNA miR-122 to regulate different aspects of HCV replication; (**b**) A structural switch in the HIV-1 5′UTR regulates the balance between genome translation and packaging. Transcripts beginning with three G residues fold into a monomer conformation and are preferentially translated. Transcripts beginning with one G residue fold into a dimer conformation. Structural switching is mediated by mutually exclusion interactions between regions U5 (pink), SL1 (blue), the AUG region (green), and a host tRNA (purple).

Hepatitis C virus (HCV) is a model of such complex RNA based regulation [85,86,87]. The *cis*-acting replicating element (CRE) in the coding region of the NS5B protein forms a long distance base pairing with the highly conserved X-region in 3′ UTR [88,89,90,91,92] (Figure 4a). This interaction is required for replication, but also acts as a regulatory switch between replication and packaging by masking the core protein binding sites present in the 3′UTR [92] (Figure 4a). At the same time, the CRE regulates HCV genome translation via a long-range intra-molecular interaction with the internal ribosome entry site (IRES) in the 5′UTR [86,93] (Figure 4a). Finally, the 3′UTR X-region contains a palindromic sequence that promotes homo-dimerization of the HCV genome via a kissing loop inter-molecular RNA-RNA interaction [85,94,95] (Figure 4a). Since homo-dimerization is incompatible with the CRE-X interaction, and because it is likely tied to the concentration of genomes and viral chaperones in the cell, this mechanism is predicted to inhibit genome replication in favour of packaging late in the replication cycle. In this way, HCV elegantly fine tunes its replication using a complex network of dynamic and mutually exclusive RNA-RNA interactions [85,86,87].

Unsurprisingly, other viruses use similar principles to regulate their replication. HIV-1 genomic RNA is transcribed by the host cell and exported into the cytoplasm as a single pool of RNA that can be either selected by the viral Gag protein for packaging into viral particles or translated by host cell ribosomes [6]. A long-standing hypothesis is that the HIV-1 5′UTR adopts two alternative structural conformations to regulate the balance between genome translation and packaging (Figure 4b). Many structural models have been proposed, but all of them have the common feature that SL1 is presented in one conformation and sequestered in another [96,97,98,99,100,101,102,103]. As previously noted, SL1 is a key packaging motif in HIV-1 because the stem of SL1 contains the major Gag binding motif [51,104]. In addition to the Gag binding site, SL1 contains a six-nucleotide palindromic loop sequence that mediates an inter-molecular kissing loop interaction leading to the formation of genome dimers [105,106,107]. Unlike HCV, which produces homodimers that remain in the cell, HIV-1 dimers are packaged into virions [108]. This process, known as dimerization, is a conserved feature of retroviral replication that is assumed, but not formally proven, to be a pre-requisite for packaging [109]. A series of NMR studies have identified a region, U5, in the 5′UTR that base pairs with the loop sequence of SL1, or alternatively with a region surrounding the AUG start site [98,100,101,110]. When the SL1 loop sequence is base paired with U5, genomic RNA is monomeric, which promotes translation (Figure 4b). When U5 is base paired with a region surrounding the AUG start codon, the SL1 loop is available for dimerization and packaging [111] (Figure 4b). Remarkably, transcription start site heterogeneity inherent to the HIV-1 promotor strongly influences the equilibrium between these two structures [98,112]. HIV-1 genomes transcribed with a single guanosine favour the dimer conformation and are packaged into viral particles, while genomes transcribed with two or three guanosines form monomers that are preferentially translated [98,112] (Figure 4b). The fact that a single GC base-pair perturbs the monomer-dimer equilibrium provides striking proof that viruses exploit metastable RNA structures in their regulation.

Added complexity comes from the fact that RNA viruses also regulate their replication using inter-molecular interactions between host RNAs and their genomes. The HCV 5′UTR contains binding sites for the host micro-RNA miR-122 [113,114,115] (Figure 4a). miR-122 is essential for the stability of HCV genomic RNA by inhibiting RNA decay by Xrn exonucleases [116,117,118]. Binding of miR-122 also increases HCV genome translation [119,120,121,122,123] and replication [124,125] by other mechanisms. Several lines of evidence suggest that miR-122 can act by inducing RNA structural changes in the 5′UTR. Specifically, miR-122 either alone or in partnership with Argonaute proteins, enhances translation by promoting the folding of a functional IRES and suppressing alternative folds of the 5′ UTR that interfere with IRES function [121,123]. Others have proposed that miR-122 enhances translation by facilitating cyclization of the genome, by promoting stranded separation of the replication intermediates, or by bringing or displacing protein co-factors to the genome [124]. Similarly, the HIV-1 5′UTR contains a binding site for a host cellular tRNALys3, which is used as a primer for reverse transcription [126,127] (Figure 4b). Its binding results in RNA conformational changes that favour dimerization, and presumably packaging [103,128].

## 4. Intermolecular RNA-RNA Interactions in Segmented Viruses

**Figure 5:**
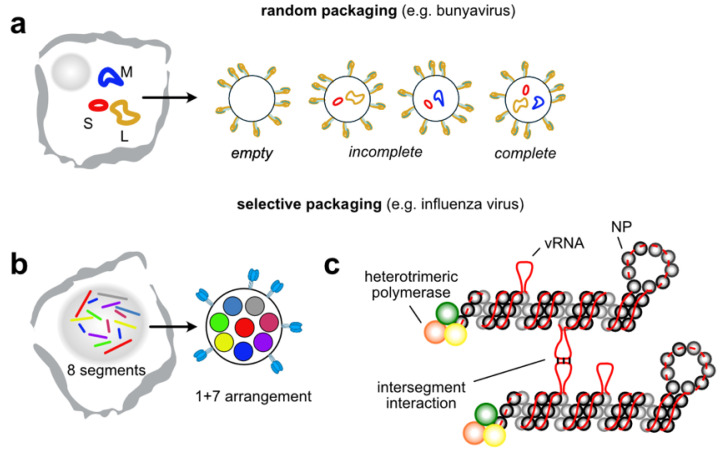
Segmented viruses package their genomes randomly or by using segment specific packaging signals. (**a**) Bunyavirus randomly package three genome segments, small (S), medium (M) and large (L), such that many progeny virions are empty or incomplete; (**b**) Influenza A virus package 8 genome segments selectively. Genome segments within budding virions are organized into a 1 + 7 arrangement with one central segment surrounded by seven others; (**c**) Influenza vRNAs are packaged as viral ribonucleoproteins (vRNPs). vRNAs are bound by nucleoprotein (NP) and a heterotrimeric polymerase. vRNA is incompletely coated by NP allowing for inter-segment vRNA-vRNA interactions to occur as a possible mechanism underlying the selective packaging process.

Many viruses split their genome into smaller independent segments. This causes problems for genome assembly, which is solved using one of two strategies: random vs selective packaging (Figure 5). Tri-segmented bunyaviruses, such as the rift valley fever virus (RVFV) and Schmallenberg virus (SBV), use the simpler strategy of random incorporation [129,130] (Figure 5a). Single molecule fluorescent in situ hybridisation (smFISH) revealed only 1 in 10 RVFV particles contain the full complement of genome segments due to the inherent heterogeneity in this packaging strategy [129]. Intuitively, as the number of segments increase, the probability of packaging one copy per particle decreases rapidly unless a large number of genome segments are incorporated per particle [131]. As this is not very efficient, many segmented viruses have overcome the genome assembly problem with specific packaging signals, which allow each distinct segment to be identified and packaged (Figure 5b).

A well-studied example is influenza A virus of the *Orthomyxoviridae* family. Its genome consists of eight negative-sense viral RNAs (vRNAs) that are packaged into viral particles as viral ribonucleoprotein (vRNP) complexes [131]. Because each vRNA encodes for an essential protein, every infectious viral particle must contain at least one copy of each segment. Indeed, smFISH experiments prove that most viral particles contain precisely one of each segment [132,133]. Furthermore, numerous electron tomography studies demonstrate that influenza vRNPs in budding viruses adopt an arrangement, also known as ‘1 + 7’ conformation, in which seven vRNPs surround a central one [134,135,136]. Altogether, these data argue for a selective packaging process. Defective interfering (DI) RNA, which naturally arise in cell culture at high multiplicity of infection (MOI), retain 100–300 nucleotides from their terminal sequences indicating that these regions contain packaging signals [137,138,139,140]. Indeed, deletion and mutagenesis studies have grossly defined terminal packaging regions within all eight vRNAs [141,142,143,144,145,146,147,148,149,150]. Terminal packaging signals are proposed to be bipartite, containing a non-specific “incorporation signal” in the UTR/promoter region, and a specific “bundling signal” in the terminal coding regions [150]. The hypothesized incorporation signal directs vRNP packaging into virions, whereas the bundling signal allows discrimination between vRNPs. The mechanism mediating this phenomenon is still not completely understood, but the most popular explanation is that packaging signals discriminate between segments by defining direct and segment specific inter-molecular RNA-RNA interactions (Figure 5c). In support of this idea, electron microscopy studies show frequent physical contacts through the entire length of each vRNP with a string-like form reminiscent of RNA [135,136,151,152], and vRNAs are able to form RNA-RNA interactions in vitro [136,153,154]. The prevailing model is that influenza vRNPs are packaged as a supramolecular complex that are held together through a network of interactions where each vRNA contacts at least one other vRNA [8]. This would help explain why mutations to packaging signals in one vRNA often affected the packaging of other vRNAs [146,149,155]. Furthermore, the capacity of RNA to tolerate mutations without disrupting structure and function would explain why packaging site mutations do not always give rise to phenotypic effects. Importantly, several vRNA-vRNA interactions have been characterised at the nucleotide level proving that at least some packaging signals define direct RNA-RNA contacts [153,156,157]. These recent results have spurred efforts to map more completely inter-segment interactions in influenza using high-throughput sequencing and RNA proximity ligation technologies [157,158]. Collectively, these studies have revealed that the inter-molecular RNA-RNA interactions are extensive, with frequent contacts seen throughout vRNAs, including in the central coding regions. Nevertheless, comprehensive maps of direct vRNA-vRNA contacts have been surprisingly difficult to interpret, with many interactions having no apparent functional role. One major conclusion could be that vRNA packaging signals are complex and redundant, but it could also reflect deficiencies in contact map technology which may miss important interactions because of known inefficiencies and biases imposed by crosslinking reagents [159] or ligation strategies [160]. Furthermore, the role of protein-RNA and protein-protein interactions in this process is not excluded. As a matter of fact, influenza nucleoprotein (NP) provides an additional layer of complexity to this process as it incompletely coats the vRNA and helps to define which vRNA sequences are available to form inter-molecular interactions [161,162,163].

Similar principles seem to apply to dsRNA segmented viruses of the *Reoviridae* family, which includes rotavirus and bluetongue virus. Rotavirus has a genome composed of eleven dsRNA segments of different sequences and lengths (0.7 to 3.1 kb) [164]. Paralleling recent results in influenza, inter-molecular interactions between segments 9, 10 and 11 of the rotavirus RNAs were observed in vitro [165]. Disruption of the putative interaction sites by mutation or with oligoribonucleotides inhibited complex formation and viral replication in cell culture [165]. Studies with bluetongue virus, which has 10 dsRNA segments, suggest a model whereby assembly begins with the formation of an initial complex built of the small RNA segments [166,167,168]. This complex would then serve as a base for sequentially recruiting the remaining RNA segments to ultimately generate a complete complex that is packaged into virions. In the case of influenza, smFISH studies reveal that sequential vRNP-vRNP interactions occur en route to the plasma membrane where packaging takes place [133,169,170]. However, current evidence indicates that there is not a single assembly pathway, but a number of alternative preferred pathways that nevertheless prevent the incorporation of more than one copy of each segment [133]. How this is achieved at the mechanistic level is still an open question.

## 5. RNA Packaging and Evolution

**Figure 6:**
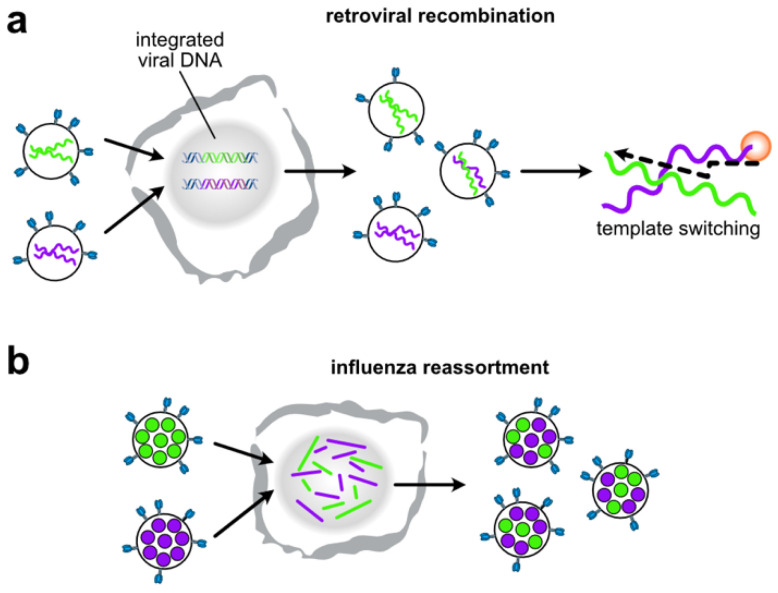
Viral RNA packaging influences viral evolution. (**a**) HIV-1 virions contain two copies of the genome. After co-infection, up to 50% of progeny co-package different genomes. Template switching during reverse transcription produce cDNA that is a chimera of the two genomes; (**b**) Influenza A virions contain eight different vRNA segments. After co-infection, reassortant progeny virions are produced containing a mixture of segments from each parental virus.

RNA based packaging signals play a much broader role in viral life cycles than assembly, and it is now appreciated that RNA virus genome structures enhance virus ‘evolvability’ by facilitating gene exchange during co-infection [171]. One widespread strategy is template switching during replication leading to recombination and the formation of genome chimeras, which is a conserved phenomenon in retroviruses [11,172] (Figure 6a). Another common strategy is genome segmentation leading to reassortment, which can be seen in rotaviruses and influenza viruses [173] (Figure 6b). Recombination and reassortment are both non-random processes that are heavily biased by RNA sequence and structure.

Retroviruses package two near identical copies of the genome as a non-covalently associated dimer [108]. One evolutionary advantage for this dimeric genome organization is that it brings together two templates for packaging into virions. Template switching during subsequent infection and reverse transcription generates a recombinant virus that is genetically distinct from the two parental viruses [174,175,176,177,178] (Figure 6a). Retroviral recombination is a major mechanism by which retroviruses escape selective pressures imposed by the immune system or antiretroviral therapy [11]. As previously noted, HIV-1 dimerization is mediated by the palindromic loop sequence of SL1 [105,106,107]. Sequence variations that are unable to form the kissing loop interaction are also defective in recombination due to their inability to be co-packaged into virions [179]. Indeed, the loop sequences in subtype B (GCGCGC), and subtypes A, C and G (GUGCAC) are incompatible. Inter-subtype recombination is thus much lower compared to intra-subtype recombination [105,179,180]. Interestingly, HIV-1 genomes containing deletions in SL1 are still packaged into virions as dimers, albeit at a lower level than wild-type viruses [181,182]. This provides strong evidence that so-far undetected inter-molecular interaction exist throughout the HIV-1 genome that may enable the formation inter-subtype recombinants even viruses are unable to form the kissing-loop interaction at SL1.

Packaging signal incompatibilities are also thought to be a major restriction to reassortment in segmented viruses [156,157,183,184,185,186]. This is especially important for influenza where introductions of sequence variation from animal reservoirs have led to pandemics in the past [187]. Fortunately, divergence in packaging signals between human and animal viruses is one of many steps that may block reassortment [184,185]. The molecular mechanism restricting reassortment probably lies in the inability of divergent sequences to form intermolecular vRNA-vRNA interactions required for packaging [156,157,183]. This provides hope that in the future, better knowledge of viral structures may be repurposed to predict or even direct viral evolution to combat both emerging and endemic RNA viruses.

## 6. Outlook

Generally, viral RNA packaging is assumed to be a process dependent on a few clearly defined RNA structural motifs specifically recognized by a viral protein. However, when evaluating binding affinities and specificities of those RNA-protein complexes in vitro, they often do not show the specificity that is observed for the packaging process in vivo [25]. Thus, it may be reasonable to think of packaging as an integrative process that involves multiple co-occurrent interactions that must also take place at the correct time and subcellular localization for genome packaging to occur.

Excitingly, methods to characterize RNA virus packaging signals are being developed and refined to better resolve the details of these integrative processes. Approaches to study viral RNA packaging spans disciplines and can now shed light on this process across multiple scales. For example, advanced cryo-EM techniques promise to determine RNA structures at high resolution in three-dimensions [188,189,190]. Furthermore, cryo-EM [191,192,193,194] and X-ray scattering [195,196] may reveal RNA-protein interaction sites inside of viral capsids. In parallel, RNA structural probing techniques are being developed that enable the detection of structural changes in RNA that may be the result of RNA packaging, e.g., by identifying alternative structures [197,198] and/or mapping RNA-protein interaction sites on the RNA [199,200]. Continual improvements in quantitative live, super resolution, and expansion microscopy will be key for understanding mechanisms of viral assembly in cells [133,201,202,203,204,205]. These improvements are beginning to reveal how inherent variability in viral assembly allow viruses to replicate and evolve in the face of complex and unpredictable environments [133,203]. Finally, comparative high throughput sequencing can identify RNA packaging signals, be it historically from identifying genomic constraints of packaging-competent defective viral genomes [206,207,208,209,210], or more recently by reverse genetics systems that quantify relative packaging efficiencies of large pools of mutants in parallel [53]. Together, these technical revolutions are sure to dramatically improve our understanding of the molecular mechanisms of viral RNA genome packaging across virus families. In the near future, these insights can be pivoted into novel antiviral drugs and vaccines for controlling these important human pathogens.
